# Split-Brain: What We Know Now and Why This is Important for Understanding Consciousness

**DOI:** 10.1007/s11065-020-09439-3

**Published:** 2020-05-12

**Authors:** Edward H. F. de Haan, Paul M. Corballis, Steven A. Hillyard, Carlo A. Marzi, Anil Seth, Victor A. F. Lamme, Lukas Volz, Mara Fabri, Elizabeth Schechter, Tim Bayne, Michael Corballis, Yair Pinto

**Affiliations:** 1 Department of Psychology, University of Amsterdam, Amsterdam, the Netherlands; 2 School of Psychology, University of Auckland, Auckland, New Zealand; 3 School of Health Sciences, University of California Dan Diego, La Jolla, CA USA; 4 School of Medicine and Surgery, University of Verona, Verona, Italy; 5 Sackler Centre for Consciousness Science, Sussex University, Brighton, UK; 6 Klinik für Neurologie, Universitätsklinikum Köln, Kerpener Str, 62 Köln, Germany; 7 Dipartimento di Medicina Sperimentale e Clinica, Via Tronto 10/A, 60020 Ancona, Italy; 8 Department of Philosophy, Washington University, St. Louis, MO USA; 9 Department of Philosophy, Monash University, Melbourne, Australia

**Keywords:** Epilepsy, Split-brain, Lateralization, Visual perception, Consciousness agents

## Abstract

Recently, the discussion regarding the consequences of cutting the corpus callosum (“split-brain”) has regained momentum (Corballis, Corballis, Berlucchi, & Marzi, *Brain*, *141*(6), e46, 2018; Pinto et al., *Brain, 140*(5), 1231–1237, 2017a; Pinto, Lamme, & de Haan, *Brain, 140*(11), e68, 2017; Volz & Gazzaniga, *Brain*, *140*(7), 2051–2060, 2017; Volz, Hillyard, Miller, & Gazzaniga, *Brain*, *141*(3), e15, 2018). This collective review paper aims to summarize the empirical common ground, to delineate the different interpretations, and to identify the remaining questions. In short, callosotomy leads to a broad breakdown of functional integration ranging from perception to attention. However, the breakdown is not absolute as several processes, such as action control, seem to remain unified. Disagreement exists about the responsible mechanisms for this remaining unity. The main issue concerns the first-person perspective of a split-brain patient. Does a split-brain harbor a split consciousness or is consciousness unified? The current consensus is that the body of evidence is insufficient to answer this question, and different suggestions are made with respect to how future studies might address this paucity. In addition, it is suggested that the answers might not be a simple yes or no but that intermediate conceptualizations need to be considered.

## Introduction

The term “split-brain” refers to patients in whom the corpus callosum has been cut for the alleviation of medically intractable epilepsy. Since the earliest reports by van Wagenen and Herren (1940) and Akelaitis (1941, 1943) on the repercussions of a split-brain, two narratives have emerged. First and foremost is the functional description, pioneered by Gazzaniga, Sperry and colleagues (Gazzaniga, Bogen, & Sperry, 1963; Gazzaniga, Bogen, & Sperry, 1962; Sperry, 1968), in which the intricacies, the exceptions, the effects of different testing conditions, and the experimental confounds have been delineated by decades of extensive research with a relatively small group of patients (Berlucchi, Aglioti, Marzi, & Tassinari, 1995; Corballis, 1994; Corballis et al., 2010; Corballis, 2003; Luck, Hillyard, Mangun, & Gazzaniga, 1989; Pinto, Lamme, & de Haan, 2017b; Volz, Hillyard, Miller, & Gazzaniga, 2018). It is important to note that even in this small group there are differences. In some patients all commissures were severed (“commissurotomy”), in others only the corpus callosum was cut (“callosotomy”) and some patients fall somewhere in between these two boundaries. Now, the search term “split-brain” results in a total of 2848 publications in the database of the Web-of-Science and 29,300 hits on Google Scholar, indicating a wealth of detailed information. The other depiction of split-brain patients entails the first-person perspective of the split-brain. In other words, “what is it like” to be a split-brain patient? It is especially this perspective that has captured the attention of the general press, popular science books and basic textbooks. By its nature, this second narrative lacks the detail of the functional description of the phenomenon, but it captures the intriguing question of how unity of consciousness is related to brain processes. Dominant in this description is the idea that in a split-brain each hemisphere houses an independent conscious agent. This notion, and particularly the concept of an isolated but conscious right hemisphere that is unable to express its feelings, desires or thinking due a lack of language, has captured the imagination (Gazzaniga, 2014).

It is important to clarify what we mean by unified consciousness. Here, we use the term in the sense of “subject unity” as defined by Bayne (Bayne & Chalmers, 2003; Bayne, 2008; Bayne, 2010). Subject unity is present if all the experiences generated in a system belong to one subject. In other words, if a system contains a first person perspective, then subject unity is preserved if that system only contains one such perspective, but subject unity is absent if the system contains multiple first person perspectives. Thus, in our definition of conscious unity, consciousness in a split-brain is split if each cortical hemisphere houses an independent conscious agent.

The view that consciousness is split in a split-brain has significantly impacted cognitive neuroscience at large. For instance, currently dominant theories about conscious awareness - the Integrated Information Theory (Tononi, 2005; Tononi, 2004) and the Global Neuronal Workspace Theory (Dehaene & Naccache, 2001; Dehaene, Kerszberg, & Changeux, 1998) - may be critically dependent on the validity of this view. Both theories imply that without massive communication between different subsystems, for instance cortical hemispheres, independent conscious agents arise. Thus, if the split consciousness view is invalid, these theories may be critically challenged.

**Fig. 1:**
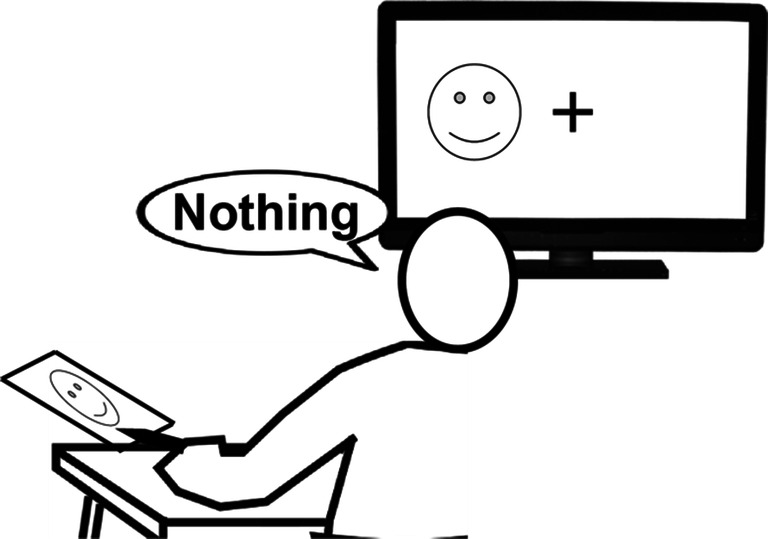
One of the most well-known split-brain findings is that the patient claims verbally not to have seen the stimulus in the left visual field, yet indicates the identity of it with their left hand. This suggests that the left hemisphere (controlling verbal output) is blind to the left visual field, while the right hemisphere (controlling the left hand) does perceive it

The idea of split consciousness in a split-brain had its origin in the early split-brain studies (Gazzaniga, 1967; Gazzaniga, 1975; Gazzaniga et al., 1962; Sperry, 1968). These studies tested patients primarily in the two perceptual domains where processing is largely restricted to the contralateral hemisphere, that is vision and touch. In these early studies, stimuli, for instance objects, that were presented to the left hemisphere either physically in the right hand or as an image in the right visual half-field, could be readily named (as the left hemisphere is dominant for language) or pointed out with the right hand (which is controlled by the left hemisphere). The patient’s behavior became intriguing when the stimuli were presented in the left visual field or in the left hand. Now the patient, or at least the verbal left hemisphere, appeared oblivious to the fact that there had been a stimulus at all but was nevertheless able to select the correct object from an array of alternatives presented to the left hand or the left visual half-field (see Fig. 1). In a particularly dramatic recorded demonstration, the famous patient “Joe” was able to draw a cowboy hat with his left hand in response to the word “Texas” presented in his left visual half field. His commentary (produced by the verbal left hemisphere) showed a complete absence of insight into why his left hand had drawn this cowboy hat. Another astonishing example involved the same patient. MacKay and MacKay (1982) flashed a digit in the left visual field and trained the patient to play a version of ‘20 questions’ across hemispheres. The left hemisphere guessed the answer vocally, and the right hemisphere provided responses by pointing ‘up’ (meaning ‘guess a higher number’) or ‘down’ with the left hand. In this way the patient managed to vocalize the right answer. This suggests two independent conscious agents communicating with each other (one steering the left hand, the other agent controlling vocal expressions). However, note that an alternative interpretation is possible. Perhaps the patient knows the answer but finds it hard to vocalize. The ‘20 questions’ then simply help him in finding the correct vocalization.

Thus, these early observations suggested that there is no meaningful communication between the two hemispheres in split-brain patients. This led to the hypothesis that there might be two separate conscious agents, a left hemisphere that is able to talk to us and can explain what it sees and feels, and a mute right hemisphere that cannot communicate in language but that can nevertheless show that it has perceived and recognized objects and words. However, over time this view has eroded somewhat due to several anomalies (even right from the start) that may challenge this view.

## Common Ground

An early observation, suggesting some remaining unification concerned what Joe Bogen called the “social ordinariness” of split-brain patients. Apart from a number of anecdotal incidents in the subacute phase following the surgery, these patients seem to behave in a socially ordinary manner and they report feeling unchanged after the operation (Bogen, Fisher, & Vogel, 1965; Pinto et al., 2017a; R. W. Sperry, 1968; R. Sperry, 1984), although their extra-experiment behavior has not been systematically observed in great detail (Schechter, 2018). While the right hemisphere appears to be better at recognizing familiarity from faces, self-face recognition, that is the ability to realise immediately that a presented photograph represents you, appears to be available equally to both hemispheres in a split-brain patient (Uddin et al., 2008; Uddin, 2011). Thus, it seems unlikely that a mute but conscious right hemisphere would not have made itself known one way or the other. Thus, right from the start a paradox arose. The controlled lab tests suggested that consciousness is split in split-brain patients. Yet, everyday experiences of the patient and their close ones suggests that only one person exists in a split-brain. Additionally, it has been suggested that the two separate consciousnesses-hypothesis presumes that in the intact brain (before surgery) both hemispheres were conscious but connected via the corpus callosum, and they only became dissociable due to the operation. This casts doubt on the viability of the two consciousness view.

Crucially, the lab tests themselves were not always supportive of the split-consciousness view. Multiple experimental results showed that capacity for communication between the hemispheres varies both across patients and across tasks. For instance, a central observation in split-brain patients concerns the inability to compare visual stimuli across the midline. In other words, when one stimulus is presented to the left visual hemifield and the other to the right hemifield, the patient cannot accurately indicate whether both stimuli are the same or not, although they can do so when both stimuli are presented within one visual field. This is consistent with the notion that each hemisphere independently perceives the contralateral visual field, and that an intact corpus callosum is necessary for integration. Although there are indeed many examples of split-brain patients who are incapable of comparing stimuli across the midline, prominent examples can also be found of patients who can compare stimuli across the midline (Johnson, 1984; but see Seymour, Reuter-Lorenz, & Gazzaniga, 1994). This points to an important problem in the field, namely, individual differences. One aspect that may be important for individual differences is handedness differences. Variations in handedness may lead to differences in language capabilities in the right hemisphere, and could even underly differences in inter-hemispheric integration.

**Fig. 2:**
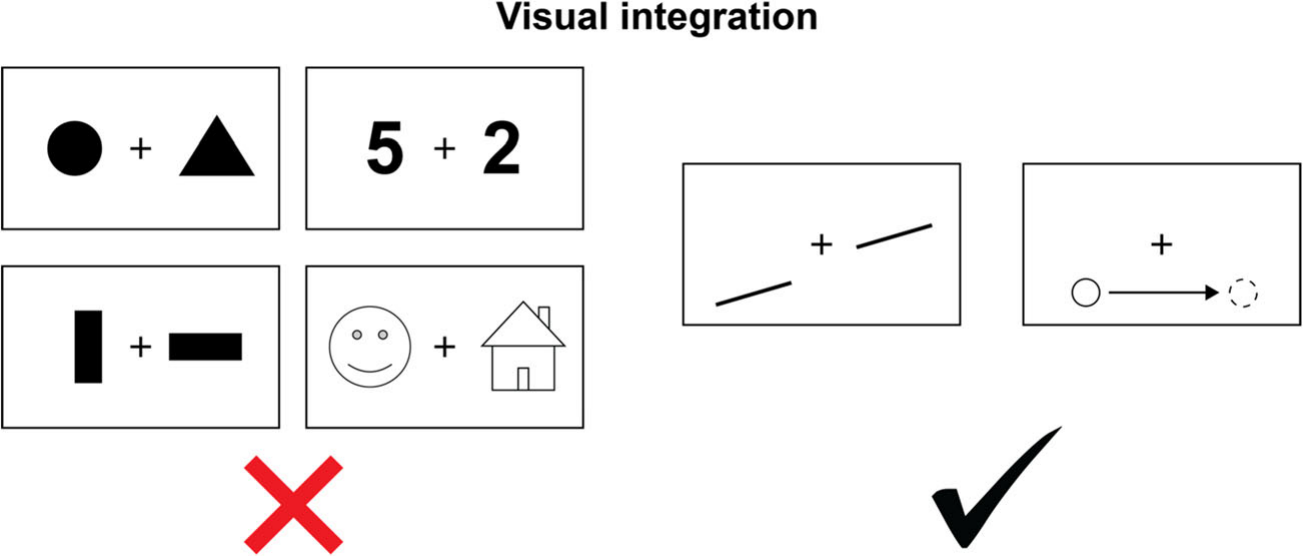
Although most split-brain patients cannot compare visual features such as shape and object identity across the midline, other features, such as good continuation of lines, and apparent motion, are integrated without a corpus callosum

Moreover, under certain circumstances nearly all tested split-brain patients seem able to compare, or integrate, particular types of stimuli across the two visual half-fields (see Fig. 2). An early demonstration of across hemifields integration is the study by Eviatar and Zaidel (1994). They showed that split-brain patients could accurately indicate the identity and shape of upper- and lower-case letters in either hemifield, regardless of with which hand they responded, for instance accurately identifying the letter A in the left visual field with the right hand. Yet these patients were mostly unable to compare these same stimuli across visual fields. In another experiment, two tilted lines were presented with a gap between them. The lines were positioned in such a way that extending them across the gap would either cause the lines to coincide or to run in parallel. When split-brain patients indicate whether the lines are parallel or coincident, they are highly accurate, even when both line segments are located in different half-fields (Corballis, 1995; Pinto, de Haan, Lamme, & Fabri, n.d.; Sergent, 1987; Trevarthen & Sperry, 1973). Another example of visual integration across the midline involves apparent motion. When two dots are presented in succession at a short distance (2 to 14 visual degrees), split-brain patients are able to accurately indicate whether the dots create apparent motion, or that they were presented simultaneously or with delays too long to provoke apparent motion. Critically, they are able to do so even when one dot appears in the left visual field, and the other in the right visual field (Knapen et al., 2012; Naikar & Corballis, 1996; Ramachandran, Cronin-Golomb, & Myers, 1986). Clearly, under specific conditions, there is meaningful communication between the two hemispheres in the absence of the corpus callosum.

Another observation that suggests some form of unity across the two visual half fields concerns detection and localization of stimulation, for instance, a brief flash (see, for example, an early study on the response times to light flashes with the ipsi- or contralateral hand: Clarke & Zaidel, 1989). Several investigations (Corballis, Corballis, Fabri, Paggi, & Manzoni, 2005; Pinto et al., 2017a; Trevarthen & Sperry, 1973) have demonstrated convincingly that split-brain patients can accurately report the presence and location of stimuli for any position in the whole visual field, with either hand, and even verbally. Accurate detection and localization appears to be possible for all patients and all stimuli (different shapes, figures, equiluminant stimuli) tested so far. Thus, when patients in earlier studies said that they saw “nothing” when a stimulus was presented in the left visual half-field, they may have meant that they could see it but that they could not identify or retrieve the name of the object.

Other findings point to a crucial difference between the degree of lateralization of visual-perceptual processing and producing overt responses. Perception appears to be more split, while responding remains largely unified. Whether a stimulus appears in the left or the right visual hemifield strongly impacts performance of split-brain patients. However, response type (left hand, right hand or verbally) seems to have a much smaller, or no effect at all. For instance, Pinto et al., 2017a) found that the split-brain patient was much better at matching pictures to sample stimuli in the left visual field. Yet, for the exact same stimuli matching pictures to verbal labels was vastly superior when the stimuli appeared in the right visual field. Crucially, response type did not play any role. The patient was better in matching pictures to sample for stimuli in the left visual field, even if they responded verbally or with the right hand. Similarly, Levy, Trevarthen, and Sperry (1972) presented split-brain patients with chimeric or composite faces, that is, one half-face in each visual field. Subsequently the patient either matched the chimeric face to sample, or attached a verbal label to it. Verbal matching was mostly based on the half-face in the right visual field, while matching to sample was mainly driven by the half-face in the left visual field. But crucially, the latter was the case, independent of whether the patient responded with the left or the right hand.

Thus, it seems that in split-brain patients perceptual processing is largely split, yet response selection and action control appear to be unified under certain conditions. This, by itself, does not prove whether a split-brain houses one or two conscious agents. One explanation could be that the split-brain houses two agents, each having their own experiences, who synchronize their behavioral output through various means. Another possible explanation is that a split-brain houses one agent who experiences an unintegrated stream of information who controls the entire body, comparable to watching a movie where sight and sound are out-of-sync. At any rate, these findings challenge the previously mentioned classic split-brain description, which is still found in reviews and text books (Gray, 2002; Wolman, 2012). In this classic characterization the patient indicates that they saw nothing when a stimulus appeared in the left visual field. Yet, to their own verbal surprise, the left hand correctly draws the stimulus. The aforementioned examples of unity in action control suggests that these effects may depend on the type and complexity of the response that is required.

## Interpretations

There are three, not-mutually exclusive, hypotheses concerning the mechanisms involved in, seemingly, preserved unity in the split-brain. The first notion is that information is transferred subcortically. The second idea is that ipsilateral motor control underlies unity in action control. The third idea claims that information transfer is based on varies forms of inter-hemispheric collaboration, including subtle behavioral cues. The first proposal (Corballis Corballis, Berlucchi, & Marzi, 2018; de Haan et al., 2019; Pinto, Lamme, & de Haan, 2017b; Pinto et al., 2017a; Savazzi et al., 2007; Mancuso, Uddin, Nani, Costa, & Cauda, 2019) suggests that the multitude of subcortical connections that are spared during surgery are responsible for the transfer of information. As was initially pointed out by Trevarthen (1968) and Trevarthen and Sperry (1973) and recently stressed by Pinto, de Haan, and Lamme (2017a) and Corballis et al. (2018), there are many commissures (white matter tracts that connect homologous structures on both sides of the central nervous system) and decussations (bundles that connect different structures on both sides) that link nuclei that are known to be involved in perceptual processing. The importance of these commisural connections for transferring visual information in split-brain patients has been highlighted by Trevarthen and Sperry (1973). Moreover, the role of these connections in a split-brain has recently been demonstrated by bilateral fMRI activations in the first somatosensory cortex, after unilateral stimulation of trunk midline touch receptors (Fabri et al., 2006) and in the second somatic sensory area after unilateral stimulation of hand pain receptors (Fabri, Polonara, Quattrini, & Salvolini, 2002). Uddin and colleagues used low-frequency BOLD fMRI resting state imaging to investigate functional connectivity between the two hemispheres in a patient in whom all major cerebral commissures had been cut (Uddin et al., 2008). Compared to control subjects, the patient’s interhemispheric correlation scores fell within the normal range for at least two symmetrical regions. In addition, Nomi and colleagues suggested that split-brain patients might rely particularly on dorsal and ventral pontine decussations of the cortico-cerebellar interhemispheric pathways as evidenced by increased fractional anisotropy (FA) on diffusion weighted imaging (Nomi, Marshall, Zaidel, Biswal, Castellanos, Dick, Uddin & Mooshagian, 2019). Interhemispheric exchange of information also seems to occur in the domain of taste sensitivity, activation of primary gustatory cortex in the fronto-parietal operculum was reported in both hemispheres after unilateral gustatory stimulation of the tongue receptors (Mascioli, Berlucchi, Pierpaoli, Salvolini, Barbaresi, Fabri, & Polonara, 2015). Note that patients may differ with respect to how many of these connections have been cut, and this might also explain some of the individual variance among patients. Moreover, in all patients subcortical structures remain intact. For instance, the superior colliculus is known to integrate visual information from both hemispheres and project information to both hemispheres (Meredith & Stein, 1986; Comoli et al., 2003). Such structures may support attentional networks, and may enable the right hemisphere to attend to the entire visual field. In turn, attentional unity could help in unifying cognitive and motor control, which may subserve ipsilateral motor control.

The second point concerns the ipsilateral innervation of the arms. Manual action is not strictly lateralized, and the proximal (but not the distal) parts of the arm are controlled bilaterally, although the ipsilateral contribution remains undetermined. This could explain why split-brain patients may respond equally well with both hands in certain experimental conditions (Corballis, 1995; Gazzaniga, Bogen, & Sperry, 1967; Pinto, de Haan, & Lamme, 2017a). First, there is substantial evidence that bilateral cortical activations can be observed during unilateral limb movements in healthy subjects. In addition, ipsilesional motor problems in arm control have been observed in patients with unilateral cortical injuries, and finally there is evidence from electrocorticography with implanted electrodes for localization of epileptic foci showing similar spatial and spectral encoding of contralateral and ipsilateral limb kinematics (Bundy, Szrama, Pahwa, & Leuthardt, 2018). While these observations argue convincingly for a role in action control by the ipsilateral hemisphere, they do not prove that a hemisphere *on it’s own* can purposefully control the movements of the ipsilateral hand. Thus, the role of ipsilateral arm-hand control in explaining split-brain findings is currently not settled.

The third hypothesis argues that in addition to whatever direct neural communication may exist between the hemispheres, they may inform one another via strategic cross-cueing processes (Volz & Gazzaniga, 2017; Volz et al., 2018). The split-brain patients underwent surgery many years prior to testing, and the separated perceptual systems have had ample time to learn how to compensate for the lack of commissural connections. For example, subtle cues may be given by minimal movements of the eyes or facial muscles, which might not even be visible to an external observer but are capable of encoding, for example, the location of a stimulus for the hemisphere that did not “see” it. A cross-cueing mechanism might also allow one hemisphere to convey to the other which one of a limited set of known items had been shown (Gazzaniga & Hillyard, 1971; Gazzaniga, 2013).

Finally, it is possible to entertain combinations of the different explanations. For instance, it is conceivable that in the subacute phase following split-brain surgery the hemispheres are ineffective in communicating with each other. During this initial phase, phenomena such as an “alien hand” - that is a hand moving outside conscious control of the (verbal) person - may be present. In the ensuing period, the patients may have learned to utilize the information that is exchanged via subcortical connections, ipsilateral motor control or cross-cueing to coordinate the processing of the two hemispheres. In such a way, the patient may counteract some of the effects of losing the corpus callosum.

## What do We Need to Know?

This paper aims to contribute to the agenda for the next decade of split-brain research. Full split-brain surgery is rare these days, and it is important that we try to answer the central questions while these patients are still available for study. In order to examine the variations between patients it would be useful to test as many of the available patients as possible with the same tests.

One important goal is to map out precisely how much functionality and information is still integrated across hemispheres in the split-brain, and what the underlying principles are. For instance, in some cases the two hemispheres seem to carry out sensory-motor tasks, such as visual search, independently from one another (Arguin et al., 2000; Franz, Eliassen, Ivry, & Gazzaniga, 1996; Hazeltine, Weinstein, & Ivry, 2008; Luck, Hillyard, Mangun, & Gazzaniga, 1994; Luck et al., 1989), while in other cases functions such as attentional blink, or attentional cueing, seem to be integrated across hemispheres (Giesbrecht & Kingstone, 2004; Holtzman, Volpe, & Gazzaniga, 1984; Holtzman, Sidtis, Volpe, Wilson, & Gazzaniga, 1981; Pashler et al., 1994; Ptito, Brisson, Dell’Acqua, Lassonde, & Jolicœur, 2009). An important challenge is to unveil why some cognitive functions can be carried out independently in the separated hemispheres while other functions engage both hemispheres. Furthermore, it is now clear that accurate detection and localization is possible across the whole visual field, and there is some evidence that even more information concerning visual images can be transferred between hemispheres. Although we have some understanding of what types of information can be transferred in the visual domain, our knowledge base in the somatosensory domain is much more limited. This is probably due to a bias throughout cognitive neuroscience and psychology, leading to a strong focus on vision in split-brain research. It is important to collect converging evidence by investigating the somatosensory system which is also strongly lateralized. Note that in somatosensory processing transfer between hemispheres (about 80% correct for the bimanual conditions) has been observed for basic same-different matching of real objects (Fabri, Del Pesce et al., 2005).

Another important goal is to obtain a more detailed description of the perceptual, cognitive and linguistic capabilities of the disconnected right hemisphere. For understanding unity of mind, two capabilities specifically are crucial. First, experiments investigating aspects of the conscious mind often go beyond simple visual processing, and future studies will thus critically depend on testing high-level cognitive abilities of both hemispheres. Specifically, language abilities, crucial for understanding questions and instructions, will likely play a pivotal role. Thus, the first question is to what extent the right hemisphere is capable of language processing. Note that complicated instructions (Gazzaniga, Smylie, Baynes, Hirst, & McCleary, 1984; Pinto et al., 2017a; Zaidel, 1983), for instance relating to mental imagery (Johnson, Corballis, & Gazzaniga, 2001; Kosslyn, Holtzman, Farah, & Gazzaniga, 1985; Sergent & Corballis, 1990), seem to be well within the reach of the right hemisphere. Moreover, right hemisphere language capabilities seem to improve over time (Gazzaniga, Volpe, Smylie, Wilson, & LeDoux, 1979; Gazzaniga et al., 1996). Longitudinal language tests (for instance with a Token test: De Renzi & Vignolo, 1962) would further illuminate the extent of right hemisphere language processing.

Second, unveiling to what extent each hemisphere is capable of subserving consciousness at all seems relevant for unity of mind as well. If the disconnected right hemisphere can produce full-blown consciousness, then questions regarding unity of mind are clearly more pertinent then if the right hemisphere only produces minimal amounts of consciousness. Right hemisphere consciousness can be studied through novel neural paradigms (Bekinschtein et al., 2009; Casali et al., 2013; Pitts, Metzler, & Hillyard, 2014; Shafto & Pitts, 2015). For instance, Bekinschtein et al. employed EEG to measure if the brain detected irregularities (as indicated by an event-related potential [ERP] signal called the P3) in different states of consciousness. They found that when consciousness was reduced, local irregularities were still detected - for instance after three high auditory tones a low tone evoked a P3. However, global irregularities - several times a low tone followed three high tones, then on the critical trial three high tones were followed by another high tone - did not evoke a P3 when consciousness was reduced. Crucially, when consciousness was unimpaired both local and global irregularities evoked a P3 response. Right hemisphere consciousness may also be studied in other patient groups where interhemispheric communication is hampered. One particularly interesting group are post-hemispherotomy patients (Lew, 2014). These patients have been surgically treated to disconnect an entire hemisphere (usually for intractable epilepsy), but unlike hemispherectomy patients the disconnected hemisphere remains in place in the cranium and remains vascularized.

Clearly, the central question, whether each hemisphere supports an independent conscious agent, is not settled yet. Novel paradigms in this respect could lead to progress. For instance, a pivotal question is whether each hemisphere makes its own decisions independent of the other hemisphere. If each hemisphere produces its own autonomous conscious agent then this should be the case. That is, if two agents are asked to freely choose a random number, then the odds that they consistently pick the same number are small. And vice versa, if each hemisphere makes its own conscious decisions, independent of the other hemisphere, then this seems to rule out unity of mind. Note that each hemisphere making its own decisions is different from information processing occurring independently per hemisphere. Unconscious information processing is almost certainly split across hemispheres in a split-brain. However, this does not prove that consciousness is split or unified. Even in a healthy brain, where consciousness is unified, many unconscious processes run independently, and in parallel.

One way to tackle the central question is by having the hemispheres respond to questions in parallel. Overt behavior most likely does not allow for this, due to bilateral motor control processes sketched earlier. However, perhaps parallel responding is possible if the hemispheres produce covert responses. For instance, the patient could be asked to pick one of four options and indicate their choice by carrying out certain content-specific mental imagery tasks. This imagery can then be decoded in parallel from each hemisphere using neuroimaging techniques (see Owen et al., 2006 for a similar approach with vegetative state patients). If each hemisphere harbors an autonomous conscious agent, then it is highly unlikely that the two hemispheres will consistently make the same choices. Thus, if the choices are uncorrelated across hemispheres, then this may critically challenge the unified mind view.

Another way to tackle the question of unified consciousness in the split-brain is to employ ERPs as markers of concurrent conscious processing in the left and right hemispheres. For instance, in one study (Kutas, Hillyard, Volpe, & Gazzaniga, 1990) visual targets were presented either separately to the left or right visual field or to both visual fields simultaneously. It was found that the P300 - a signal possibly reflecting conscious processing of a visual target (Dehaene & Changeux, 2011; Dehaene, Charles, King, & Marti, 2014; Salti, Bar-Haim, & Lamy, 2012) - was reduced for bilateral targets. This suggests some type of integration of conscious processing. Studies employing ERPs may indicate whether conscious processing is unified, while unconscious processing is split, which would be suggestive of unified consciousness.

## Conclusions

In summary, the pivotal issue in split-brain research is whether dividing the brain divides consciousness. That is, do we find evidence for the existence of one, or two conscious agents in a split-brain? Note that intermediate results may be found. Perhaps some measures indicate unified consciousness while others do not. This would then provoke further interesting questions on the unity of consciousness. What are the crucial measures for unity of consciousness? If intermediate results are found, more unconventional possibilities should be entertained as well. For instance, although difficult to fathom, some philosophers have suggested that a split-brain does not contain one or two observers, but a non-whole number of conscious agents (Nagel, 1971; Perry, 2009), for instance one and a half first-person perspectives. If evidence for this position is found, then its implications would stretch beyond split-brain patients. It would suggest that our intuitions on the indivisibility of the experiential self may be mistaken. One way to think of this is as with the difference between conscious and unconscious processing. Perhaps this is not a dichotomous distinction, but a continuum between more or less conscious. Similarly, perhaps the existence of a first-person perspective is not dichotomous, but gradual as well. Another possibility is that a split-brain does contain a whole number of conscious agents, but that consciousness across these agents is only partially unified. That is, the agents share some conscious experiences and decisions, but not all (Lockwood, 1989; Schechter, 2014; Schechter, 2018; Schechter, 2013). Finally, another way to look at this is in terms of ‘dissociation’, as in depersonalization (Phillips et al., 2001; Sierra et al., 2002). Perhaps the number of agents is not altered, but the agent feels depersonalized in some situations, and therefore no longer feels that they control the actions, or even experience the information, that has just occurred in their brain.

New findings on the unity of consciousness in a split-brain could fundamentally impact currently dominant consciousness theories. Global Neuronal Workspace Theory (Dehaene & Naccache, 2001; Dehaene et al., 1998) asserts that consciousness arises when information that is processed in unconscious (or preconscious) modules is broadcast to a central ‘workspace’, primarily residing in frontal regions of the brain. Although not very explicit on the unity of consciousness in split-brain patients, Global Neuronal Workspace Theory seems to endorse the split consciousness idea, given that each hemisphere has its own prefrontal hub, enabling broadcasting of whatever information is processed in that hemisphere.

Integrated Information Theory (Tononi, 2005; Tononi, 2004) has specifically addressed the issue of split brain (for instance in Tononi, 2004). Integrated Information Theory asserts that ‘phi’, a measure of how integrated information is, determines the level of consciousness. The higher phi, the more conscious a system is. Moreover, local maxima in phi correspond to conscious agents. If in a system all subsystems are highly interconnected, then phi is highest for the system as a whole, and local maxima are absent. Thus, such a system produces only one conscious agent. However, if subsystems only exchange minimal amounts of information, then phi per subsystem is higher than phi for the system as whole. In such a case each subsystem creates its own conscious agent. In a split-brain, connectedness, that is integration of information, is much higher within than across hemispheres. Therefore, according to Integrated Information Theory consciousness should be split in a split-brain.

Recurrent Processing theory (Lamme & Roelfsema, 2000; Lamme, 2004; Lamme, 2010) argues for the independence of consciousness from attention, access, or report. This theory has addressed the issue of report specifically, making the case that consciousness and reportability, whether verbal or manual, should be viewed as entirely independent (Lamme, 2006; Tsuchiya, Wilke, Frässle, & Lamme, 2015). Crucially, this theory states that even in the normal mind, ‘islands’ of unattended yet conscious information reside (Lamme, 2006). In these cases, all the information, although functionally unintegrated, is nonetheless experienced by the same mind. Support for this view comes from findings in multiple object tracking (Pinto, Howe, Cohen, & Horowitz, 2010; Pinto, Scholte, & Lamme, 2012; Pylyshyn & Storm, 1988). Here, evidence indicates that when moving objects in two visual hemifields are tracked, attention is split (Howe, Cohen, Pinto, & Horowitz, 2010) and each hemisphere processes the relevant information in the contralateral visual field independently of the other (Alvarez & Cavanagh, 2005; Cavanagh & Alvarez, 2005; Drew, Mance, Horowitz, Wolfe, & Vogel, 2014). That is, the left hemisphere only tracks the right visual field and vice versa. Yet, although the visual information is not integrated across hemispheres, from a first person perspective, it seems clear that the subject experiences all moving objects across the entire visual field. Another example of the dissociation between consciousness and reportability is the so-called partial report paradigm (Pinto et al., 2017b; Pinto, Sligte, Shapiro, & Lamme, 2013; Sligte, Scholte, & Lamme, 2008; Sperling, 1960). In these paradigms subjects seem to remember more than they can report. Thus, reportability and consciousness are dissociated. Perhaps in split-brain patients this dissociation is simply more pronounced. That is, consciousness remains unified, but reportability has become more dissociated, thereby inducing the appearance of two independent agents. In sum, according to the Recurrent Processing theory, integration of information is not needed for a unified mind, implying that the mind may remain unified when the brain is split. Thus, different theories of consciousness have different predictions on the unity of mind in split-brain patients, and await the results of further investigation into this intriguing phenomenon.
